# First-line anti-VEGF plus EGFR-TKI in EGFR-mutant NSCLC: adding the ARTEMIS trial to the puzzle of current evidence

**DOI:** 10.1038/s41392-021-00813-y

**Published:** 2021-12-06

**Authors:** Susanne Hafner

**Affiliations:** Institute of Pharmacology of Natural Products and Clinical Pharmacology, Ulm University, D-89081 Ulm, Germany

**Keywords:** Outcomes research, Lung cancer, Drug development

In the ARTEMIS-CTONG1509 multicenter phase 3 study recently published in Cancer Cell, Zhou et al. investigate the combination of the EGFR tyrosine kinase inhibitor (TKI) erlotinib and anti-angiogenic bevacizumab in treatment-naive Chinese patients with advanced EGFR-mutant non-small cell lung cancer (NSCLC).^1^ The primary endpoint of progression-free survival (PFS) was significantly extended from 11.2 months with erlotinib monotherapy to 17.9 months with the combination.

## Evidence of bevacizumab plus TKI in advanced EGFR-mutant NSCLC

**Fig. 1:**
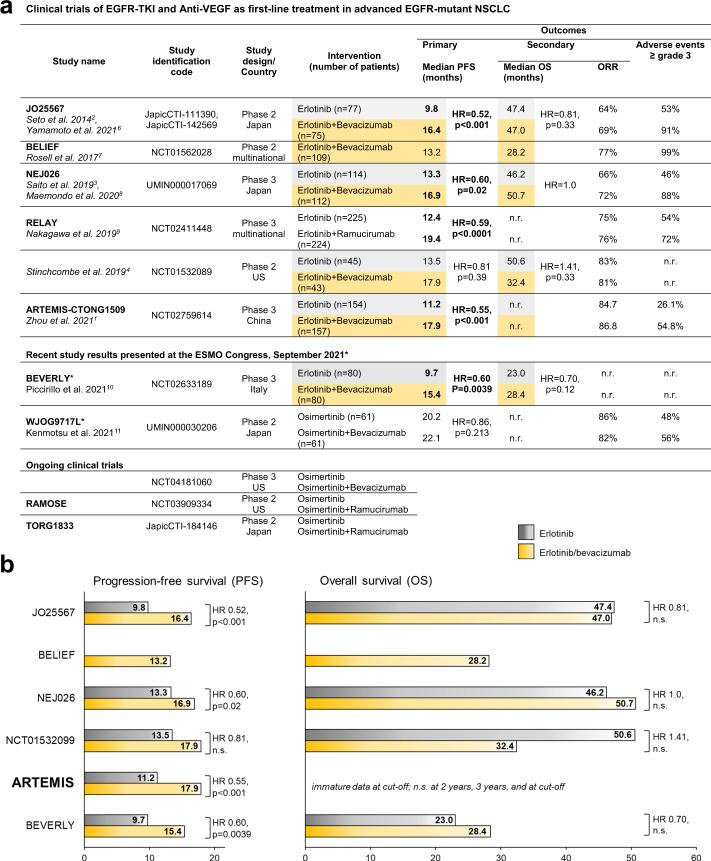
Clinical trials of EGFR-TKI and Anti-VEGF as first-line treatment in advanced EGFR-mutant NSCLC. **a** Overview on the completed and ongoing clinical trials of EGFR-TKI and Anti-VEGF as first-line treatment in advanced EGFR-mutant NSCLC. Modified and updated from: Chen et al. 2020^5^; data were extracted from the indexed publications and/or publically available clinical trial databases; bold values indicate significant differences; grey and yellow colors highlight study data for PFS and OS with erlotinib and erlotinib+bevacizumab treatment, which are illustrated in (**b**). *Data presented at the European Society for Medical Oncology (ESMO) Conference, September 2021; PFS refers to investigator-assessed PFS in the BEVERLY trial and blinded independent radiologic reviewer-assessed PFS in the WJOG8717L trial, respectively. EGFR epidermal growth factor receptor, PFS progression-free survival, ORR objective response rate, OS overall survival, n.r. not reported, TKI tyrosine kinase inhibitor^6–11^. **b** Progression-free survival (left panel) and overall survival (right panel) in prospective phase 2 and 3 clinical trials of EGFR-TKI erlotinib and bevacizumab as first-line treatment in advanced EGFR-mutant NSCLC.

In advanced NSCLC, the addition of bevacizumab to platin-based chemotherapy improved overall survival from 10.3 to 12.3 months and led to the approval as first-line strategy in advanced NSCLC in 2006. Since then, the implementation of molecular testing and availability of further targeted (immuno-)therapies has fundamentally changed the therapeutic approach in advanced-stage NSCLC. Most cases of NSCLC with driver mutations of the EGFR (up to 20% of advanced NSCLC) initially respond well to EGFR-TKI, but develop resistance to TKI over time. In 2014, the JO25567 phase 2 trial showed that the addition of bevacizumab to EGFR-inhibitory erlotinib improved PFS from 9.8 to 16.4 months in Japanese patients.^2^ Further evidence of prolonged PFS in a Japanese population was provided by the phase 3 NEJ026 trial in 2019.^3^ However, another phase 2 study conducted in the United States including mainly non-Asian patients did not find an improvement for PFS in spite of comparable study design.^4^ Likewise, the BELIEF trial performed in 8 European countries reported shorter PFS and overall survival time compared to the trials performed in Asian populations. Since a review by Chen et al. in 2020,^5^ further evidence has become available—see Fig. 1 for an updated comparison between PFS and secondary outcomes in previous trials and in the ARTEMIS trial. EGFR-mutant NSCLC is much more frequent in Asian populations, and substantial differences in etiopathology, response to therapy, and clinical outcome between Asian and non-Asian patients have been observed. The ARTEMIS trial shows PFS improvement by additional bevacizumab in Chinese patients and confirms the previous findings of JO25567 and NEJ026. At the recent ESMO Conference, the primary results of the BEVERLY phase 3 trial were presented showing an improvement of PFS with erlotinib plus bevacizumab in Italian patients as well (Fig. 1a). Thus, regardless of ethnic origin, additional bevacizumab provides a benefit in terms of PFS versus erlotinib monotherapy.

## Does additional bevacizumab improve overall survival?

Although the overall survival data of the ARTEMIS trial are immature at cut-off date, there is no benefit at the interim analysis (37.9 months with dual therapy versus 41.1 months with erlotinib alone). Nowadays, most clinical trials in NSCLC are powered for PFS as the primary endpoint, and differences between PFS and OS are commonly observed. However, the ultimate goal remains to prolong a patient’s life at good quality. We should bare in mind that PFS is a surrogate parameter and relies on imaging-based assessment of disease progression according to RECIST. Bevacizumab leads to the regression of existing tumor-associated vasculature and inhibition of new blood vessel growth, and this might change blood flow and the appearance of target lesions in imaging. Consequently, reduced enhancement of contrast agent might not directly correlate to tumor response, baring the risk of overestimation of treatment response.

Beyond the confirmation of previous data on PFS, Zhou et al. explore the molecular mechanisms of acquired resistance to TKI therapy: At disease progression, EGFR T790M was detected in 41% of patients in the erlotinib plus bevacizumab arm and 61% of patients treated with erlotinib alone. This shows that—contrary to previous hypotheses—anti-VEGF therapy does not inhibit the development of TKI resistance. Treatment tolerability is another concern: Even though in the ARTEMIS trial high-grade adverse events occurred less frequently in comparison to previous studies, additional bevacizumab undoubtedly increases the risk of high-grade toxicities about twice and thereby negatively impacts treatment safety (Fig. 1a). Thus, major hurdles for long-standing therapeutic success have not been overcome yet.

## Benefit for patients with brain metastasis

Whereas preexisting CNS metastasis had been an exclusion criterion in JO25567, the ARTEMIS trial suggests that patients with intracranial disease might benefit even more from the addition of bevacizumab, in particular regarding overall survival (31.6 months with dual therapy versus 26.8 months with erlotinib monotherapy, *p* = 0.052). Bevacizumab is known to reduce the need of corticosteroids in brain tumor patients, and this was attributed to stabilization of the blood–brain barrier and reduction of vasogenic edema and thus tumor mass effects. However, Zhou et al. do not report on the administration of corticosteroids in either study arm, which might be an indicator of a similar mode of action in NSCLC with CNS metastasis.

Meanwhile, the third-generation TKI osimertinib has shown superiority over erlotinib and gefitinib in terms of PFS, OS, and quality of life, and is preferred for first-line therapy of advanced EGFR-mutant NSCLC. However, hopes on the combination of osimertinib with VEGF inhibitors are dampened by the recently presented data of the phase 2 Japanese WJOG9717L study showing no improvement of PFS by addition of bevacizumab to osimertinib (Fig. 1a). Against the background of significantly higher brain exposure of osimertinib in comparison to other TKI in preclinical in vivo models, and strong evidence for osimertinib efficacy in CNS metastasis provided by the FLAURA trial, patients with CNS metastasis at baseline might profit even more from osimertinib plus VEGF inhibitors. The corresponding subgroup analyses of ongoing trials are awaited with interest.

The ARTEMIS trial confirms previous evidence that the combination of bevacizumab and erlotinib significantly prolongs PFS in advanced EGFR-mutant NSCLC. However, caution in data interpretation and clinical implementation is advisable because the clinical benefit in comparison to osimertinib is unclear and an impact on overall survival and other patient-relevant endpoints has not been shown thus far.

## References

[1] Zhou, Q. et al. Bevacizumab plus erlotinib in Chinese patients with untreated, EGFR-mutated, advanced NSCLC (ARTEMIS-CTONG1509): a multicenter phase 3 study. *Cancer Cell* **39**, 1279–1291.e1273 (2021). 10.1016/j.ccell.2021.07.005 34388377

[2] Seto T (2014). Erlotinib alone or with bevacizumab as first-line therapy in patients with advanced non-squamous non-small-cell lung cancer harbouring EGFR mutations (JO25567): an open-label, randomised, multicentre, phase 2 study. Lancet Oncol..

[3] Saito H (2019). Erlotinib plus bevacizumab versus erlotinib alone in patients with EGFR-positive advanced non-squamous non-small-cell lung cancer (NEJ026): interim analysis of an open-label, randomised, multicentre, phase 3 trial. Lancet Oncol..

[4] Stinchcombe TE (2019). Effect of erlotinib plus bevacizumab vs erlotinib alone on progression-free survival in patients with advanced EGFR-mutant non-small cell lung cancer: a phase 2 randomized clinical trial. JAMA Oncol..

[5] Chen F, Chen N, Yu Y, Cui J (2020). Efficacy and safety of epidermal growth factor receptor (EGFR) inhibitors plus antiangiogenic agents as first-line treatments for patients with advanced EGFR-mutated non-small cell lung cancer: a meta-analysis. Front. Oncol..

[6] Yamamoto, N. et al. Erlotinib plus bevacizumab vs erlotinib monotherapy as first-line treatment for advanced EGFR mutation-positive non-squamous non-small-cell lung cancer: survival follow-up results of the randomized JO25567 study. *Lung Cancer* **151**, 20–24 (2021). 10.1016/j.lungcan.2020.11.020 33279874

[7] Rosell, R. et al. Erlotinib and bevacizumab in patients with advanced non-small-cell lung cancer and activating EGFR mutations (BELIEF): an international, multicentre, single-arm, phase 2 trial. *Lancet Respir*. *Med*. **5**, 435–444 (2017). 10.1016/S2213-2600(17)30129-7 28408243

[8] Maemondo, M. et al. NEJ026: Final overall survival analysis of bevacizumab plus erlotinib treatment for NSCLC patients harboring activating EGFR-mutations. *J*. *Clin*. *Oncol*. **38**, 9506 (2020).

[9] Nakagawa, K. et al. Ramucirumab plus erlotinib in patients with untreated, EGFR-mutated, advanced non-small-cell lung cancer (RELAY): a randomised, double-blind, placebo-controlled, phase 3 trial. *Lancet Oncol*. **20**, 1655–1669 (2019). 10.1016/S1470-2045(19)30634-5 31591063

[10] Piccirillo, M.C. et al. Bevacizumab + erlotinib vs erlotinib alone as first-line treatment of pts with EGFR mutated advanced non squamous NSCLC: final analysis of the multicenter, randomized, phase III BEVERLY trial. In *European Society for Medical Oncology (ESMO) Congress 2021; September 16–21, 2021. Abstract 1207O* (2021).

[11] Kenmotsu, H. et al. Primary results of a randomized phase II study of osimertinib plus bevacizumab versus osimertinib monotherapy for untreated patients with non-squamous non-small cell lung cancer harboring EGFR mutations: WJOG9717L study. In *European Society for Medical Oncology (ESMO) Congress 2021; September 16–21, 2021. Abstract LBA44* (2021).

